# Is there evidence that *Kudoa septempunctata* can cause an outbreak of acute food poisoning?

**DOI:** 10.4178/epih.e2017004

**Published:** 2017-01-30

**Authors:** Young-Bae Chung, Jong-Myon Bae

**Affiliations:** 1 Department of Parasitology, Jeju National University School of Medicine, Jeju, Korea; 2 Department of Preventive Medicine, Jeju National University School of Medicine, Jeju, Korea

**Keywords:** Etiology, Virulence, Food parasitology, Parasitic intestinal diseases, Myxozoa

## Abstract

After publishing results of a study that revealed diarrheagenic and emetic activity in 4-5-day old mice infected with *Kudoa septempunctata* (*K. septempunctata*) spores, the Korea Centers for Disease Control and Prevention reported 11 events of “*Kudoa* food poisoning” in 2015. The epidemiological design of the previous study was descriptive rather than analytical; therefore, this study aimed to further investigate the pathogenicity of *K. septempunctata*. Academic articles showing evidence of the pathogenicity of *K. septempunctata* were searched via PubMed using the citation discovery tool. Information regarding the kinds of experimental animals and inoculum spores used, as well as study results were extracted. Four articles evaluating the pathogenicity of Myxospran parasites were selected; the first article suggested the pathogenicity of *K. septempunctata*, while the remaining three articles reported no abnormal symptoms or histopathologic changes. Our findings indicate that there is weak evidence supporting the pathogenicity of *K. septempunctata*. Further studies evaluating the pathogenicity of *K. septempunctata* are needed urgently.

## INTRODUCTION

A new parasite was discovered in aquacultured olive flounders (*Paralichthys olivaceus*) in Jejudo, South Korea (hereafter Korea), in 2010 and was named *Kudoa septempunctata* (*K. septempunctata*) [1]. In December 2012, the Ministry of Health, Labour and Welfare in Japan announced that *K. septempunctata* is the causative agent of acute food poisoning and thereafter named the outbreak of the foodborne disease “*Kudoa* food poisoning” [2,3].

In 2015, Centers for Disease Control and Prevention in Korea conducted tests for *K. septempunctata* infection using vomit and fecal samples of patients with acute food poisoning symptoms who had a history of sliced, raw olive flounder consumption. released case reports on “*Kudoa* food poisoning”, detailing its characteristics [4]. The case reports identified *K. septempunctata* as the causative agent based on the fact that it was detected in each of 11 events analyzed. Table 1 shows a summary of the main epidemiological characteristics of patients with food poisoning possibly attributed to *K. septempunctata*.

However, since case reports are descriptive rather than analytic epidemiologic methods, which suggest hypotheses only instead of identifying causes, respectively, the results of the above-mentioned case reports cannot prove *K. septempunctata* as the causative agent of an acute outbreak of “*Kudoa* food poisoning.” The results do however, show that among cases with acute food poisoning, the 18S ribosomal RNA gene (rRNA) of *K. septempunctata* was detected [3]. The current study aimed to summarize the evidences regarding the pathogenicity of *K. septempunctata*, found in *P. olivaceus* of Jejudo origin, which has been reported as the causative agent of an outbreak of acute food poisoning.

## EVALUATION OF THE RELATED EVIDENCES

A PubMed citation discovery tool was used to select information from “cited articles” and “similar articles” related to the pathogenicity of *K. septempunctata* [5]. From the article by Kawai et al. [2], which first reported on the pathogenicity of *K. septempunctata* to cause acute food poisoning, a list of 11 cited articles and 115 similar articles was obtained through the PubMed search on December 11, 2016.

Using the list, we examined whether the related abstracts dealt with the pathogenicity of *K. septempunctata* to cause an outbreak of acute food poisoning; 2 [6,7] of the 11 cited articles and 1 [8] of the 115 similar articles were selected. Therefore, a total of four articles, including the article by Kawai et al. [2] were included to summarize the evidence of the pathogenicity of *K. septempunctata*. The types and numbers of experimental animals and inoculum spores, and the findings from the four articles were summarized in Table 2.

With the exception of the article by Kawai et al. [2], which reported on the pathogenicity of *K. septempunctata*, the articles [6-8] did not report any cases of diarrhea and vomiting. Although the subjects and experimental methods in the study by Jang et al. [7] were the same as those in the study by Kawai et al. [2], the results differed. Kawai et al. [2] were Japanese and claimed that the pathogenicity of *K. septempunctata* caused the outbreak of acute food poisoning; the authors of the other studies were Chinese and Korean.

## CONCLUSION AND SUGGESTION

Upon examination of the 4 studies, it was determined that the results of the study in Japan supported the pathogenicity of *K. septempunctata* to cause an outbreak of acute food poisoning, while the studies in Korea and China opposed it. Kawai et al. [2] reported that diarrhea was induced in 4-5-day-old mice infected with *K. septempunctata*; however, the remaining studies used the same methodology and showed conflicting results [7,8]. Overall, our findings suggest that the evidence for the pathogenicity of *K. septempunctata* is still weak, in light of the currently available literature. There is a lack of evidence to use the term “*Kudoa* food poisoning,” and the pathogenicity of *K. septempunctata* should be reconsidered. There is an urgent need for an international forum to discuss these discrepant results.

Discrepant results have been reported for the intestinal permeability of *K. septempunctata* in addition to its pathogenicity. In 2013, Ohnishi et al. [9] reported that *K. septempunctata* invaded the human intestinal epithelia monolayer, whereas Ahn et al. [6] did not observe such a phenomenon. Additionally, this topic should also be discussed in detail at an international forum.

In 2016, Kasai et al. [10] reported that three Kudoa species, including *K. septempunctata*, are present in fish in natural waters, in addition to aquacultured *P. olivaceus*. The intermediate hosts of *K. septempunctata* have not been identified until now and studies that consider host lifecycle are needed.

To date, the experimental evidence of the pathogenicity of *K. septempunctata* is inconsistent; there is a lack of scientific validity to support the epidemiologic causality between *K. septempunctata* and acute food poisoning based solely on the results of case reports. Hill’s criteria are used widely as the standard measure of epidemiologic causality [11]; however, it is not feasible to apply the criteria to case studies with no control group. This is supported by the epidemiologic finding in which it was argued that the measles, mumps, and rubella vaccination was associated with autism in case reports of 12 pediatric cases with autism [12]. This event had great repercussions on infection prevention policies; however, it was concluded that there was no association between the vaccination and autism [13]. Further analytic and epidemiologic studies, such as case-control studies or cohort studies, will provide evidence for evaluating epidemiologic causality in future.

In conclusion, the authors of the present manuscript highlight that the present argument about the pathogenicity of *K. septempunctata* as the cause of an outbreak of acute food poisoning is solely based on the results of animal experiments with low reproducibility. Additionally, they do not support the argument that *K. septempunctata* does not cause food poisoning.

## Figures and Tables

**Table 1.** Epidemiological characteristics of cases with food poisoning (n=94) detected by *Kudoa septumpunctata* in 2015, in Korea^1^

Characteristics	n (%)
Symptom occurrence (%)	
Mean (range)	61 (21-100)
Incubation time (hr)	
Mean (range)	2 (1-15)
Clinical symptoms^2^	
Diarrhea	78 (82.9)
Vomiting	78 (82.9)
Abdominal pain	60 (63.8)
Nausea	56 (59.5)
Chillness	55 (58.5)
Fever	35 (37.2)
Headache	6 (6.0)

1 Source from: Korea Centers for Disease Control and Prevention. Case reports of Kudoa food poisoning in 2015; 2016 [4].

2 Responses are not mutually exclusive.

**Table 2.** Summary of the evidence related to the pathogenicity of Myxosporean parasites

First author (public year) [Reference]	Experimental animal	Inoculum spores	Inoculation route	Outcome	Results
Kawai (2012) [2]	4-5-d old ddY mouse	*Kudoa septempunctata*	Oral	Bowel movements and FAR	Showing diarrheagenic activity (11/17) and significantly higher FAR
Ahn (2015) [6]	6-wk old BALB/c mice	*Kudoa septempunc-tata*	Oral	Bowl movements and histo-pathological examination	No significant diarrhea (0/32) and histopathologic changes (0/24)
Jang (2016) [7]	4-5-d old ddY mouse	*Kudoa septempunc-tata*	Oral	Bowel movements and FAR	No watery stool form (0/20) and no significant variations in FAR
Guo (2015) [8]	4-5-d old BALB/c suckling mice	*Myxobolus hongh-uensis*	Oral	Bowel movements, FAR, and histopathology	No significant change of FAR, no abnormal stool form (0/25) and no evidence of inflammation (0/25)

ddY, Deutschland, Denken, and Yoken; FAR, fluid accumulation ratio; BALB/c, albino, laboratory bread strain of the house mouse.
