# Supplementary material for: Is there evidence that Kudoa septempunctata can cause an outbreak of acute food poisoning?
Source: Epidemiol Health. 2017 Jan 30;39:e2017004. doi: 10.4178/epih.e2017004 (PMC5343106; doi:10.4178/epih.e2017004)
Supplement: Supplementary file 1 [file epih-39-e2017004-supplementary.pdf]

<Perspective>

칠성쿠도아충이 급성 식중독 원인충이란 근거는?

Is there evidence that *Kudoa septempunctata* can cause an outbreak of acute food poisoning?

정영배 [1)], 배종면 [2)]

1) 제주대학교 의학전문대학원 기생충학교실; 2) 예방의학교실

Young-Bae Chung[1)] Jong-Myon Bae[2)]

1) Department of Parasitology; 2) Department of Preventive Medicine, Jeju National University School of Medicine; Jejudo, Korea

Abstract

After publishing results of a study that revealed diarrheagenic and emetic activity in 4-5-day old mice infected with *Kudoa septempunctata* (*K. septempunctata)* spores, the Korea Centers for Disease Control and Prevention reported 11 events of "*Kudoa* food poisoning" in 2015. The epidemiological design of the previous study was descriptive rather than analytical; therefore, this study aimed to further investigate the pathogenicity of *K. septempunctata*. Academic articles showing evidence of the pathogenicity of *K. septempunctata* were searched via PubMed using the citation discovery tool. Information regarding the kinds of experimental animals and inoculum spores used, as well as study results were extracted. Four articles evaluating the pathogenicity of Myxospran parasites were selected; the first article suggested the pathogenicity of *K. septempunctata*, while the remaining 3 articles reported no abnormal symptoms or histopathologic changes. Our findings indicate that there is weak evidence supporting the pathogenicity of *K. septempunctata*. Further studies evaluating the pathogenicity of *K. septempunctata* are needed urgently.

Keyword: Fish parasitology, Virulence, Food parasitology, Parasitic intestinal diseases, Myxozoa

서론

2010년 한국 제주도에서 양식된 광어 (olive flounder)에서 발견한 원충을 *Kudoa septempunctata* (이후 칠성쿠도아충) 명명하였고 [1], 이후 2012년 12월 일본의 보건후생성 (Ministry of Health, Labour and Welfare of Japan)은 칠성쿠도아충이 급성 식중독을 일으키는 원인충이며, 이로 인한 식중독 발생을 'Kudoa food poisoning'으로 발표하였다 [2,3].

한국의 질병관리본부는 2015년부터 급성 식중독 증상을 보인 사례 중, 광어회 섭취력을 가진 사례에 대하여는 토사물 혹은 배설물에 대한 칠성쿠도아충 검사를 시행하여, 2015년 쿠도아충 사례조사 및 특성을 발표하였다 [4]. 환자 사례조사 (cases reports) 기술에 있어 분석한 11건의 발생사례 모두에서 칠성쿠도아 충이 검출되었다는 사실에 따라, '원인균이 칠성쿠도아충으로 확인'되었다고 기술하고 있으며, 표 1은 주요 역학적 특성을 정리한 것이다.

그러나 인과규명용 분석역학연구가 아니라 가설제시용 기술역학연구의 방법론인 환자사례 결과는 칠성쿠도아충을 원인균으로 단정할 수 없으며, 오직 급성 식중독 사례 중 칠성쿠도아충 18S ribosomal RNA gene (rDNA) [3]이 검출된 사례라고 해석하는 것이 정확하다. 그렇다면 제주산 광어에서 발견되는 칠성쿠도아충이 급성 식중독을 일으키는 병인 원충이라는 주장과 관련한 병인성 (pathogenicity) 근거들을 정리하여 향후 연구의 방향을 제시하는 것이 본 원고의 목적이다.

근거평가

칠성쿠도아충의 병인성에 관련한 논문을 선정하기 위해서 PubMed가 제공하는 인용논문 (cited articles)과 유사논문 (similar articles)의 정보를 활용하는 citation discovery tool을 사용하였다 [5]. 즉, 2012년에 처음으로 병인성을 주장한 Kawai et al [2]의 논문에 있어, 2016년 12월 11일 검색일에 PubMed 에서는 11편의 인용논문과 115편의 유사논문의 목록을 확보하였다.

이들 목록을 활용하여 초록을 중심으로 칠성쿠도아충의 병인성을 다루었는가를 검토하였다. 그 결과, 11편의 인용논문에서 2편 [6,7]을, 115편의 유사논문에서 1편 [8]을 각각 선정하였다. 따라서 칠성쿠도아충의 병인성의 근거를 정리할 대상은 Kawai et al. [2] 논문을 합쳐서 총 4편이었다. 선정한 4편에 있어, 실험동물의 종류 및 마리 수, 투입한 원충 종류, 그 결과를 표 2로 정리하였다.

제일 먼저 병인성이 있다고 주장한 Kawai et al. [2] 논문을 제외하고는 나머지 3편 [6-8]은 설사나 구토 현상을 확인할 수 없다고 보고하였다. 특히 Jang et al. [7]의 실험 대상 및 방법은 Kawai et al. [2]과 똑같이 수행되었지만, 그 결과는 정반대 이었다. 특이한 점은 병인성을 주장한 저자는 일본국적인 반면, 나머지는 중국과 한국이었다.

결론 및 제언

칠성쿠도아충의 병인성에 관한 4편의 논문을 살펴본 결과, 일본의 논문은 병인성을 지지하지만, 중국과 한국의 논문은 병인성을 의심하는 결과를 보고하였다. 즉, Kawai et al. [2]은 생후 4-5일 된 마우스를 사용하여 칠성쿠도아충을 주입 시 설사를 유발한다고 보고하였지만, 같은 실험을 한 결과 정반대의 결과가 도출되었다 [7,8] 이런 사실들은 칠성쿠도아충이 병인성이란 주장의 근거가 아직 미약하다. 다시 말해서 현재의 근거로는 '쿠도아충 식중독'이란 용어를 적용하기에는 근거가 부족하여서, 칠성쿠도아충의 병인성을 재고할 필요가 있다. 우선적으로는 실험결과가 상충되는 것에 대하여 국제 심포지엄을 열어 집중적으로 논의할 장을 마련할 필요가 있겠다.

병인성 뿐만 아니라 장 표피 침투성에 있어서도 상반된 결과들이 보고되었다. 2013년 Ohnishi et al. [9] 은 칠성쿠도아충은 인체 장 표피를 침투한다고 보고하였지만, Ahn et al. [6] 은 그런 현상을 관찰하지 못하였다고 보고하였기 때문이다. 이 또한 심포지엄을 개최하여 집중적으로 논의할 필요가 있다.

2016년 Kasai et al. [10]는 양식광어가 아닌 자연계 어류에서도 칠성쿠도아충을 포함하여 3 종류의 쿠도아충이 있다고 보고하였다. 현재까지 쿠도아충의 중간숙주가 무엇인지 밝혀지지 않은 가운데, 쿠도아충의 자연사에 대한 연구도 필요하겠다.

이상의 내용들을 요약한다면, 칠성쿠도아충의 병인성에 대한 실험실적 근거에 일관성이 없다는 것이다. 이렇게 실험실적 근거가 불충분한 상황에서, 오직 환자사례 결과들만으로 칠성쿠도아충을 급성식중독의 역학적 인과성 (epidemiologic causality)을 부여하는 것은 과학적 타당성이 떨어진다. 오늘날 Hill's criteria 가 역학적 인과성을 부여하는 기준으로 널리 활용되고 있는데 [11], 대조군이 없는 사례조사만으로는 이를 적용하기 어렵다. 12명의 환아 사례에 근거하여 홍역-볼거리-풍진 백신접종이 자폐증 (autism)을 일으킨다는 주장 [12]으로 전세계 감염예방정책에 큰 파장을 일으켰지만, 결국 관련이 없는 것으로 최종 결론이 났던 역학적 사건을 경험했기 때문이다 [13]. 향후 환자-대조군 연구 혹은 코호트 연구처럼 분석역학연구의 근거들이 축적되어 역학적 인과성을 평가할 근거가 생성되기를 기대한다.

마지막으로 저자들은 현 시점에서 쿠도아충의 병인성 주장은 재현성이 떨어지는 동물실험의 결과에만 근거하고 있다는 상황을 지적하면서, 또한 칠성쿠도아충이 식중독을 일으키지 않는다는 주장을 지지하는 것이 아님을 분명히 한다.

References

1. Matsukane Y, Sato H, Tanaka S, Kamata Y, Sugita-Konishi Y. *Kudoa septempunctata* n. sp. (Myxosporea: Multivalvulida) from an aquacultured olive flounder (*Paralichthys olivaceus*) imported from Korea. Parasitol Res 2010;107(4):865-72.

2. Kawai T, Sekizuka T, Yahata Y, Kuroda M, Kumeda Y, Iijima Y, et al. Identification of *Kudoa septempunctata* as the causative agent of novel food poisoning outbreaks in Japan by consumption of *Paralichthys olivaceus* in raw fish. Clin Infect Dis 2012;54(8):1046-52.

3. Sugita-Konishi Y, Sato H, Ohnishi T. Novel foodborne disease associated with consumption of raq fish, olive flounder (*Paralichthys olivaceus*). Food Safety 2014;2(4):141-50.

4. Centers for Disease Control and Prevention in Korea. Case reports of Kudoa food poisoning in 2015; 2016 [cited 2016 Dec 11]. Available from: http://cdc.go.kr/CDC/notice/CdcKrTogether0302.jsp?menuIds=HOME001-MNU1154-MNU0005-MNU0088&cid=71877 (korean)

5. Bae JM, Kim EH. Citation discovery tools for conducting adaptive meta-analyses to update systematic reviews. J Prev Med Public Health 2016;49:129-133.

6. Ahn M, Woo H, Kang B, Jang Y, Shin T. Effect of oral administration of *Kudoa septempunctata* genotype ST3 in adult BALB/c mice. Parasite 2015;22:35.

7. Jang Y, Ahn M, Bang H, Kang B. Effects of *Kudoa septempunctata* genotype ST3 isolate from Korea on ddY suckling mice. Parasite 2016;23:18.

8. Guo Q, Jia L, Qin J, Li H, Gu Z. Myxozoans and Our Dinner Table: Pathogenicity Studies of *Myxobolus honghuensis* (Myxosporea: Bivalvulida) Using a Suckling Mice Model. Foodborne Pathog Dis 2015;12(8):653-60.

9. Ohnishi T, Kikuchi Y, Furusawa H, Kamata Y, Sugita-Konishi Y. *Kudoa septempunctata* invasion increases the permeability of human intestinal epithelial monolayer. Foodborne Pathog Dis 2013;10(2):137-42.

10. Kasai A, Li YC, Mafie E, Sato H. New host records of monacanthid fish for three *Kudoa* spp. (*K. septempunctata*, *K. thyrsites*, and *K. shiomitsui*) prevalent in the olive flounder (*Paralichthys olivaceus*), with the description of *K. parathyrsites* n. sp. from a black scraper (*Thamnaconus modestus*). Parasitol Res 2016;115(7):2741-55.

11. Rothman KJ, Greenland S. Causation and causal inference in epidemiology. Am J Public Health 2005;95(Suppl 1):S144-50.

12. Wakefield AJ, Murch SH, Anthony A, Linnell J, Casson DM, Malik M, et al. Ileal-lymphoid-nodal hyperplasia, non-specific colitis, and pervasive developmental disorder in children. Lancet 1998;351:637-

41.

13. Robertson DJ, Sandler RS. Measles virus and Crohn's disease: a critical appraisal of the current literature. Inflamm Bowel Dis 2001;7(1):51-57.

Table 1. Epidemiological characteristics of cases of food poisoning (n=94) detected *Kudoa septumpunctata* in 2015, Korea*

Incidence proportion (%)		
	mean	61
	ranges	21 - 100
Incubation time (hr)		
	mean	2
	ranges	1 - 15
Clinical symptoms**		
	Diarrhea	78 (82.9%)
	Vomiting	78 (82.9%)
	Abdominal pain	60 (63.8%)
	Nausea	56 (59.5%)
	Chillness	55 (5835%)
	Fever	35 (37.2%)
	Headache	6 (6.0%)

* Sources: Centers for Disease Control and Prevention in Korea [4]

** Duplicated responses

Table 2. Summary tables for evidences related to pathogenicity of Myxosporean parasites

Author (public year) [Reference]	Experimental animal	Inoculum spores	Inoculating route	Outcome	Results
Kawai (2012) [2]	4-5-day old ddY mouse	*Kudoa septumpunctata*	oral	bowel movements and FAR	Showing diarrheagenic (11/17) activity and significantly higher FAR
Ahn (2015) [6]	6-week old BALB/c mice	*Kudoa septumpunctata*	oral	bowl movements and histopathological examination	No significant diarrhea (0/32) and no histo-pathological change (0/24)
Jang (2016) [7]	4-5-day old ddY mouse	*Kudoa septumpunctata*	oral	bowel movements and FAR	No watery stool form (0/20) and no significant variations in FAR
Guo (2015) [8]	4-5-day old BALB/c suckling mice	*Myxobolus honghuensis*	oral	bowel movements, FAR and histopathology	No significant change of FAR, no abnormal stool form (0/25) and no evidence of inflammation (0/25)

FAR (fluid accumulation ratio)
